# Aligned Nanotopography Promotes a Migratory State in Glioblastoma Multiforme Tumor Cells

**DOI:** 10.1038/srep26143

**Published:** 2016-05-18

**Authors:** Alexander Beliveau, Gawain Thomas, Jiaxin Gong, Qi Wen, Anjana Jain

**Affiliations:** 1 Department of Biomedical Engineering, Worcester Polytechnic Institute, Worcester, MA, USA; 2 Department of Physics, Worcester Polytechnic Institute, Worcester, MA, USA

## Abstract

Glioblastoma multiforme (GBM) is an aggressive, Grade IV astrocytoma with a poor survival rate, primarily due to the GBM tumor cells migrating away from the primary tumor site along the nanotopography of white matter tracts and blood vessels. It is unclear whether this nanotopography influences the biomechanical properties (i.e. cytoskeletal stiffness) of GBM tumor cells. Although GBM tumor cells have an innate propensity to migrate, we believe this capability is enhanced due to the influence of nanotopography on the tumor cells’ biomechanical properties. In this study, we used an aligned nanofiber film that mimics the nanotopography in the tumor microenvironment to investigate the mechanical properties of GBM tumor cells *in vitro*. The data demonstrate that the cytoskeletal stiffness, cell traction stress, and focal adhesion area were significantly lower in the GBM tumor cells compared to healthy astrocytes. Moreover, the cytoskeletal stiffness was significantly reduced when cultured on aligned nanofiber films compared to smooth and randomly aligned nanofiber films. Gene expression analysis showed that tumor cells cultured on the aligned nanotopography upregulated key migratory genes and downregulated key proliferative genes. Therefore, our data suggest that the migratory potential is elevated when GBM tumor cells are migrating along aligned nanotopographical substrates.

Glioblastoma multiforme (GBM) is an aggressive malignant brain tumor that accounts for 45.6% of primary brain tumors^1^. Although standard clinical treatments, such as surgical resection, chemotherapy, and radiation therapy have demonstrated to be effective, the median survival time is not significantly improved and remains at 14.6 months^2^. Moreover, the recurrence rate remains high (~90%) due to the highly invasive nature of the GBM cells^3^. In addition, cancer initiating cells (CICs), a self-renewing subset of the heterogenic tumor cell population, are highly migratory, invasive, and are responsible for recurrence of the tumor^4^. It has been shown that the GBM cells migrate and invade healthy brain tissue along white matter tracts and blood vessels^5^,^6^. However, it has yet to be elucidated whether this biological phenomena is due to the biochemical or biomechanical cues provided by these structures. It is critical to understand why these tumor cells migrate along these topographical paths in order to develop therapies to inhibit the migration of the GBM tumor cells from the primary tumor mass.

Cellular biomechanics are responsible for a variety of biological functions in eukaryotic cells, including migration, differentiation, morphogenesis, and proliferation^7^,^8^. Specifically, these processes are largely dependent on the cytoskeleton structure and its response to the surrounding extracellular matrix (ECM). Cells adhere to the local substratum via integrins, which cluster together leading to the recruitment of proteins necessary for the formation of focal adhesions and stress fibers^9^. Topographic organization of the ECM plays a key role in directing cell behavior by providing three-dimensional cues to the cell^10^.

The cytoskeleton and ECM are drastically altered in brain tumors. The actin filaments of cancer cells are transformed and their adhesion to the surrounding ECM is modified. Upon oncogenic transformation, tumor cells secrete proteases to degrade and remodel the surrounding ECM. On the intracellular level, the Rho family of GTPases activate signaling pathways to rearrange the cytoskeleton with actin-rich membrane protrusions, which include lamellipodia, filopodia, and invadopodia, along the leading edge of the cell. Activation of these pathways also lead to the assembly of stress fibers and actomyosin contraction^11^,^12^,^13^,^14^. The remodeling of the ECM and formation of the actin-rich membrane protrusions affect the cells’ deformation, altering their ability to stretch and contract, thereby abetting cellular invasion by allowing cells to migrate through tissues much faster than normal cells^15^. The cytoskeletal stiffness of tumor cells has been previously shown to correlate with the migratory and invasive potential in a variety of cancer types, including GBM, ovarian, breast, prostate, and bladder^16^,^17^,^18^,^19^,^20^,^21^,^22^,^23^. In addition, the tumor microenvironment, including nanotopography and substrate stiffness, has played a key role in the biomechanical, proliferative, and migratory properties of GBM cells^24^,^25^,^26^,^27^,^28^,^29^,^30^,^31^.

It is difficult to understand the invasive nature of GBM tumor cells without a comparable *in vitro* model that is able to recapitulate the complex *in vivo* tumor microenvironment. While the ideal approach would be to use an *in vivo* tumor model, limitations with current technology do not allow for monitoring at the microscopic, single cell level. In addition, traditional *in vitro* models quantify migration using rigid two-dimensional (2D) substrates, which do not provide a true assessment of tumor invasion due to their lack of nanotopography and relevant substrate stiffness. Although 3D hydrogels have been used to model GBM migration due to similar stiffness and chemical composition as the tumor ECM, this system lacks the nanotopographical features, which are important to GBM cytoskeletal and migration potential^32^,^33^. By developing an *in vitro* model that mimics the *in vivo* microenvironment, systematic studies may be completed to better evaluate the molecular mechanisms responsible for tumor cell migration as well as the cellular responses to the topographic cues. Jain *et al.* previously fabricated a thin film made of aligned electrospun polycaprolactone nanofibers that mimicked the physical cues provided by the white matter tracts and blood vessels and showed that intracortical tumor cells on the film were predominantly in a migratory state than proliferative state^24^. In addition to modeling GBM migration^24^,^25^,^26^,^27^,^28^,^30^, electrospun nanofibers have also been used as a model for breast cancer cell invasion^34^ and embryonic myogenesis^35^.

In this study, we investigated the mechanical differences between healthy glial cells and GBM tumor cells, together with determining how the alignment and nanotopography of the nanofibers affect the tumor cell response in terms of their migration/invasion potential. As seen in other cancer types, more invasive, malignant tumor cells were softer than less invasive tumor cells and their respective healthy, non-mutated cells. To our knowledge, investigating the invasive potential in relation to cytoskeletal stiffness for GBM tumor cells has not been previously reported. In addition, by using an aligned nanofiber film to mimic the white matter tracts and blood vessels, we demonstrated that nanotopography affected cellular biomechanics. By examining the cytoskeletal stiffness, cytoskeletal organization, and gene expression of GBM cells cultured on aligned nanofibers, randomly aligned nanofibers, smooth film, and tissue culture polystyrene (TCPS), we identified substrate topography is correlative with the GBM tumor cells’ propensity to be in a more migratory or proliferative state.

## Results and Discussion

In this study, we investigated how the cytoskeletal mechanical properties of GBM tumor cells correlate to their migration potential. Additionally, we analyzed whether the cytoskeletal mechanical properties altered based upon the alignment and nanotopography of the substrates. Our data showed that the more invasive GBM tumor cells were the more compliant they were. In addition, the more invasive cells exerted less traction forces than the primary astrocytes that have lower invasive potential. Furthermore, when seeded on an aligned nanotopographic substrate that mimicked the *in vivo* tumor microenvironment, cytoskeletal stiffness further decreased and an increased expression of migratory related genes were observed, suggesting that substrate nanotopography and alignment have an effect on the mechanisms involved in GBM invasion.

### Greater Cytoskeletal Stiffness Observed in Astrocytes than in GBM Tumor Cells

**Figure 1:**
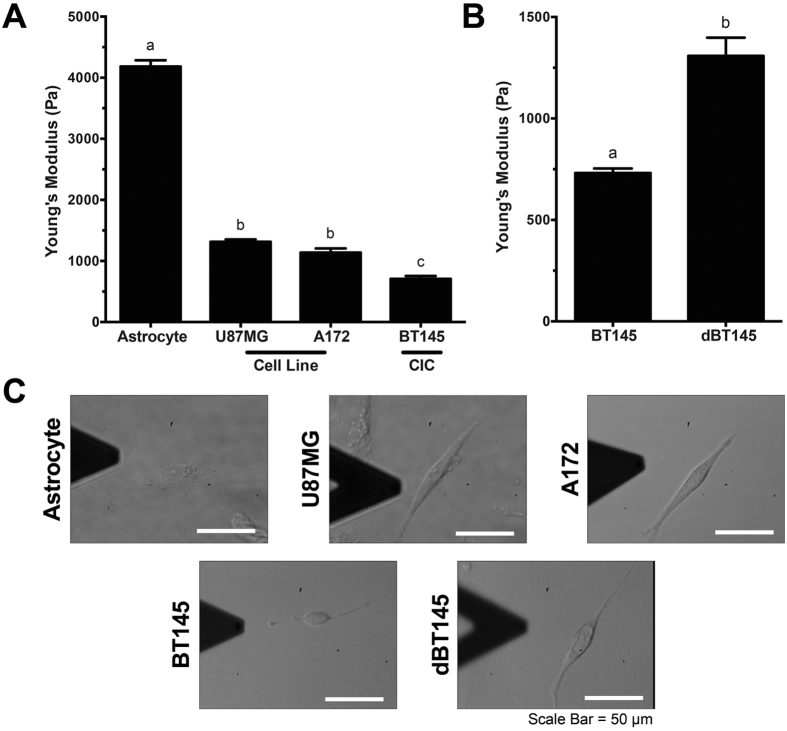
Cytoskeletal stiffness of cells decreases with increasing invasive potential. Atomic force microscopy was used to determine stiffness of primary astrocytes and GBM tumor cells when plated on collagen coated TCPS. (**A)** Cytoskeletal stiffness of primary rat astrocytes, GBM cell lines, U87MG and A172, and primary GBM CICs, BT145. Analysis showed that GBM tumor cells were significantly softer than healthy astrocytes (p < 0.0001). Further, highly invasive CICs were significantly less stiff than less invasive GBM cell lines (p < 0.05). (**B)** After differentiation CICs with FBS, stiffness of cells significantly increased 2-fold (p < 0.01). (**C)** Representative brightfield images of astrocytes and tumor cells. N = 3, mean ± SEM.

As GBM is categorized as a Grade IV astrocytoma, the difference in cytoskeletal stiffness between GBM tumor cells and non-cancerous healthy primary astrocytes was measured using atomic force microscopy (AFM). The cytoskeletal stiffness was tested on two GBM cell lines (U87MG and A172), and primary GBM CICs (BT145). Conventional thought is that primary GBM tumor cells were derived directly from genetically mutated astrocytes or glial precursor cells (i.e. EGFR amplification/mutation, PTEN loss/mutation, etc.)^36^. Therefore, primary rat post-natal day 2 astrocytes and mouse neural stem cells were used as the non-cancerous, healthy cells. Average stiffness measurements and representative images for each cell type are shown in Fig. 1. Astrocytes were significantly stiffer than each GBM tumor cell type, with an average stiffness of 4184 ± 102.3 Pa (p < 0.0001). There was no statistical difference between the two GBM cell lines, U87MG and A172 tumor cells, which had an average stiffness of 1315 ± 39.98 Pa and 1138 ± 68.58 Pa, respectively. Primary GBM CICs, BT145, were statistically less stiff than the primary astrocytes and the GBM tumor cell lines (p < 0.01), with an average stiffness of 653.3 ± 35.37 Pa (Fig. 1A). Finally, NSCs had a similar stiffness to the CICs when plated on laminin (data not shown). Morphologies of the cells were also noticeably different between the various cell types (Fig. 1D). Astrocyte morphology was more spread on the TCPS compared to the spindled morphology exhibited in the tumor cells.

As a healthy cell undergoes oncogenic mutations, many cellular attributes are altered resulting in the abnormal growth and migratory behavior of cells^15^. Due to the role of the cytoskeleton on cell migration, previous studies identified biophysical attributes, specifically cytoskeletal stiffness, as a biomarker for invasive potential in a variety of cancers. Tumor studies using AFM to measure cytoskeletal stiffness have shown that more invasive breast, ovarian, and prostate cancer cells are less stiff than their less invasive, benign, or healthy cell counterparts^18^,^20^,^37^. Significant reductions in stiffness, 2–5 fold, were observed between immortalized ovarian surface epithelial cells and different ovarian cancer cell lines^20^. In addition, Andolfi *et al.* showed that high and low grade glioma stem cells are less stiff than the non-tumorigenic glioma-associated-stem cells^23^. Similar results were observed in our experiments with the GBM tumor cells, as the highly invasive CICs were more compliant than the U87MG and A172 tumor cell lines, which together, were significantly softer than non-cancerous astrocytes. During invasion, cancer cells need to deform their bodies in order to conform to the surrounding tissue and migrate. The reduced stiffness indicates a greater capability to deform within the aggressive tumor cells, which facilitates the migration and invasion through the surrounding ECM leading to secondary tumor sites^15^. In addition, NSCs have also been shown to be highly migratory within the brain. Due to their enhanced migratory capacity toward cancerous/diseased tissue, these cells have been researched as a tool to deliver therapies to tumor cells^38^,^39^.

### Cellular Traction Stress Greater in Astrocytes than GBM Tumor Cells

**Figure 2:**
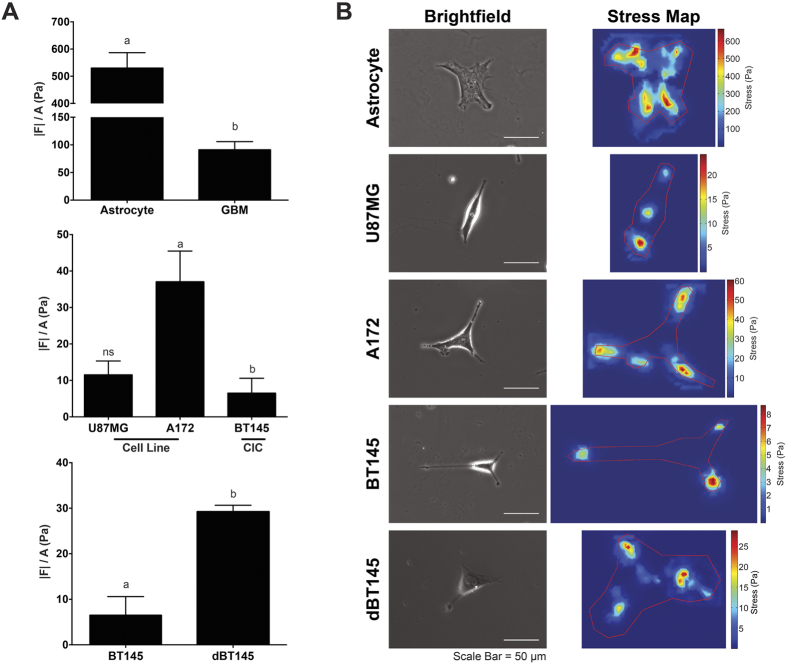
Cell traction forces decrease with increasing cell invasive potential. (**A**) Cell traction force (|F|) is normalized to its projected cell area (A) to determine average traction stress (|F|/A) for each cell. *Top panel:* TFM analysis demonstrated that average cell traction stresses between healthy astrocytes and GBM tumor cells significantly decrease (p < 0.0001), nearly 83% when seeded on an 8% acrylamide gel. *Middle panel:* When GBM tumor cells are separated based on cell type on a 5% acrylamide gel, there was a significant difference (p < 0.05) in stresses generated between tumor cell line A172 and CIC cells, as CIC’s generated 84% less stresses than A172 cells. No differences were observed between U87MG tumor cells and other cells. *Bottom panel*: Upon differentiation of CICs, traction force significantly increases (p < 0.01) when compared to their non-differentiated counterparts. (**B)** Representative brightfield and stress maps of astrocytes and tumor cells. Stronger stresses are observed throughout the entire cell body of astrocytes. Weaker stresses, however, are observed on tumor cells. Furthermore, these stresses are primarily polarized. N = 3, mean ± SEM.

Average cell traction stress of the primary astrocytes, U87MG, A172, and BT145 were measured using traction force microscopy (TFM) (Fig. 2). Cells were cultured overnight on polyacrylamide gels before being measured. For the primary astrocytes, a stiffer gel was used to measure the cell traction forces as the softer gels used for the tumor cells were too compliant and were pulled by the extremely contractile astrocytes, thus causing the beads to be in a different focal plane than the rest of the gel. This resulted in inaccurate measurements (data not shown). Therefore, a stiffer gel using 8% acrylamide and 0.1%-bis-acrylamide was used for the astrocytes. As the BT145 tumor cells did not adhere well or generate traction on the aforementioned gel, the BT145 tumor cells were cultured on a 5% acrylamide and 0.08%-bis-acrylamide gel. As gel stiffness influences the traction force generated, U87MG and A172 tumor cells were plated on both the 8% and 5% gel, and compared with the traction forces from the astrocytes and BT145 tumor cells, respectively. Cell traction forces were normalized to respective projected cell area to determine average cell traction stress.

Figure 2A illustrates that non-cancerous astrocytes exerted significantly greater cell traction stress on the 8% acrylamide gel compared to the malignant GBM tumor cells. The average cell traction stress exerted by the astrocytes was 530 ± 56.5 Pa compared to 91.2 ± 14.7 Pa exerted by the malignant GBM tumor cells (p < 0.0001). When comparing the traction stresses of the tumor cell lines and primary CICs, the A172 tumor cells exerted significantly greater stress on the 5% acrylamide gel compared to BT145 cells, with an average cell traction stress of 37.1 ± 8.41 Pa and 6.51 ± 4.08 Pa, respectively (p < 0.05). However, a statistical difference was not observed between the U87MG tumor cells (11.5 ± 3.78 Pa) and the A172 or BT145 tumor cells. As traction forces were normalized to respective cell area, the differing traction forces are not due to differences observed in cell area. Figure 2B displays representative stress maps of the cells. For the non-cancerous astrocytes, the maximum traction stresses reaches as high as 775 Pa; however, the maximum traction stresses of malignant tumor cells are an order of magnitude smaller.

Cellular traction forces have also been investigated in other cancers to demonstrate how differing traction stresses can correlate to the tumor cells’ invasive potential; however, conflicting results have been reported. Similar to what was observed in this study, an inverse relationship between traction stress and cell metastatic potential in breast cancer cells was observed^40^. Similarly, MacKay *et al.* treated GBM tumor cell lines with constitutively active (CA) RhoA and found that the increased expression in RhoA led to increased cell traction forces and decreased migration, which was probably due to the tumor cells adhering too strongly to the substratum^29^. However, other studies that investigated transformed fibroblasts, breast, prostate, and lung cancer cells, showed the opposite trend, as these cell types exerted significantly more total cell traction force than their non-metastatic counterparts^41^,^42^. One potential reason for these conflicting results is the differing native microenvironments between the different cell types. The brain has a highly specialized ECM. In addition to being considerably softer than other tissues, the brain has a lower percentage of fibrous proteins and an abundance of hyaluronic acid compared to other tissues^32^. Cell types will likely respond to varying substrates differently. It has been previously reported that substrate stiffness affects both migration speed and traction forces in a biphasic manner, with an optimal stiffness that promotes the migration speed varying between different cell types, suggesting that migration and traction forces may be linked due to the cells’ innate environment^43^.

### Actin and Focal Adhesion Analysis of Astrocytes and GBM Tumor Cells Demonstrate that Tumor Cells Exhibit Reduced Focal Adhesion Presence

**Figure 3:**
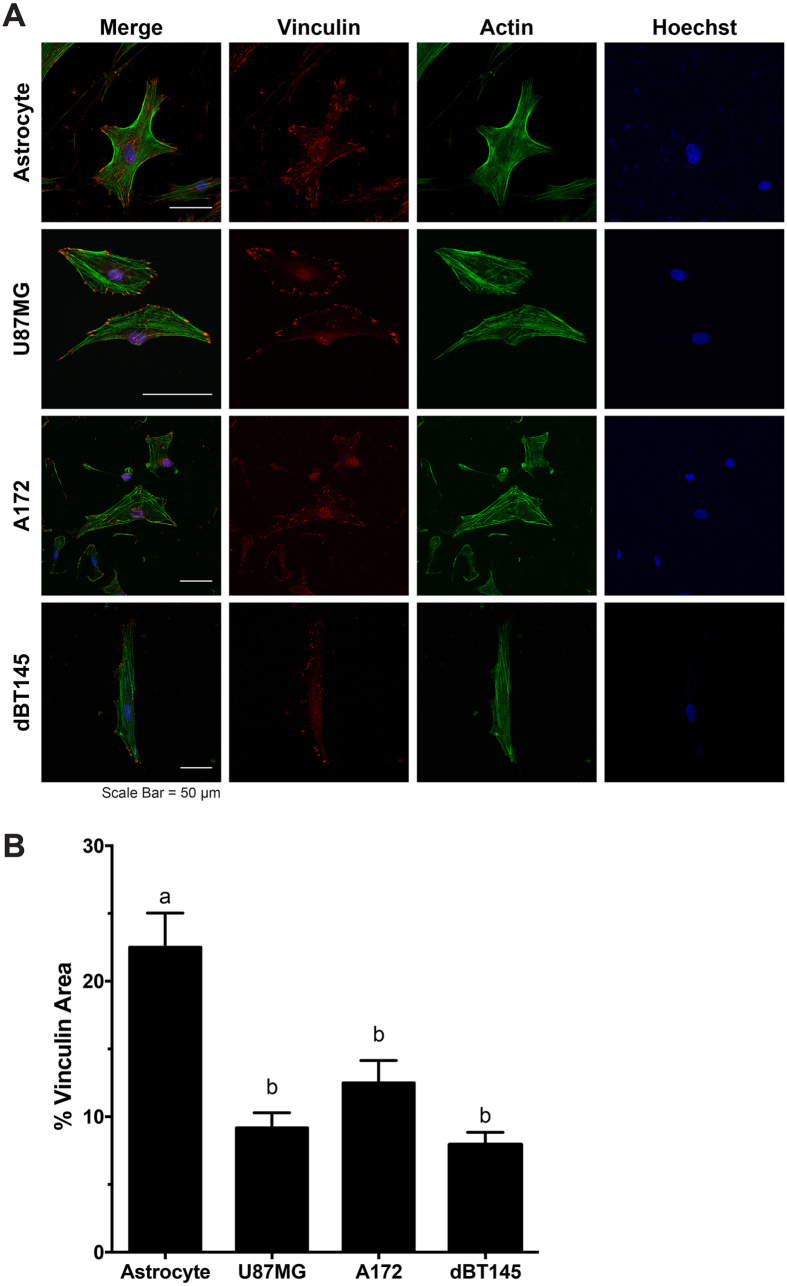
Cytoskeletal organization of astrocytes and tumor cells by staining for vinculin, a focal adhesion protein, and F-actin. (**A**) Representative images of astrocytes and tumor cells of vinculin (red), F-actin (green), and counterstained with Hoechst (blue). Densely organized actin networks were observed in astrocyte cultures; however disorganized actin filaments were observed in U87MG and A172 tumor cell cultures. Further, vinculin was largely was localized throughout the entire cell body of astrocytes, however primarily localized to the periphery of tumor cells. (**B**) Quantification of focal adhesions demonstrated that tumor cells resulted in significant reduction (p < 0.05) in vinculin area/cell area compared to healthy astrocytes. N = 3, mean ± SEM.

To determine if there were differences in cytoskeletal organization between astrocytes and malignant tumor cells, cells were stained for vinculin (red), a focal adhesion protein, as well as F-actin (green) (Fig. 3). The fluorescent images of the vinculin and F-actin co-stain can be seen in Fig. 3A. Astrocytes exhibited aligned, densely-packed stress fiber networks distributed throughout the cell body, while actin distribution in GBM tumor cells appeared less dense. As marked differences were noted in focal adhesion formation, the percentage of positive vinculin area within a cell was quantified for the astrocytes and the GBM cell lines, U87MG, and A172. As astrocytes displayed a larger spreading area than the tumor cells, the ratio of vinculin area to cell area provides a reliable normalized measurement. As seen in Fig. 3B, astrocytes exhibited significantly larger focal adhesion areas (22.5 ± 2.52%) than the U87MG (4.36 ± 1.12%) and A172 (12.5 ± 1.66%) tumor cells (p < 0.05). A statistical difference in the percentage of positive vinculin area between the two GBM cell lines, U87MG and A172, was not observed. This reduction in focal adhesion area also provides reason as to why the GBM tumor cells had weaker cell traction forces. The primary BT145 CICs were stained for vinculin and actin; however, an observable amount of vinculin was not positively stained, which may be due to the CICs being a non-adherent tumor sub-population and vinculin not being localized to the focal adhesions.

The differing biomechanical properties between the cancer and non-cancer cells are largely due to the changes in the cytoskeleton organization. Similar to what was observed in this study, it has been shown that non-malignant breast and ovarian cells formed a dense network of parallel actin filaments distributed throughout the cell body, however, their cancerous counterparts formed fewer filaments, most of which were shorter and largely disorganized, leading to the reduction in cytoskeletal stiffness^18^,^20^. Focal adhesion complexes play a significant role in cell migration, as well as adhesion to the substratum. When focal adhesions attach and pull the surrounding ECM along the leading edge, traction forces are generated within the cell, moving the cell forward. In conjunction with focal adhesion formation, there must be a turnover of focal adhesion attachment at the trailing edge to allow for continuous directional cell migration^9^. Due to its role in migration, overexpression of vinculin has been shown to reduce cancer cell migration and invasion. Conversely, a lack of vinculin expression has been associated with the development of many cancers, and potentially plays a role in epithelial-mesenchymal transition (EMT), thus suggesting a potential reason for the reduction of vinculin in the GBM cells^44^.

### Analysis of Biomechanical Properties Correlate CIC Differentiation with Decreased Invasive Capabilities

CICs, which are highly migratory and mainly responsible for tumor recurrence, are a key target for cancer therapies. As these cells are resistant to traditional chemotherapeutic strategies, differentiation therapy is being developed as an approach to treat GBM tumors to inhibit their stem-like phenotype thereby reducing tumorigenicity. By inducing differentiation in stem-like primary glioma cells, Campos *et al.* showed a decrease in cellular invasion^45^. While the majority of other studies focus on the biochemical mechanism of CIC differentiation, we investigated the effect of differentiation on the biophysical properties of these cells.

To determine if the difference in biophysical properties between the GBM cells lines and the GBM CICs was due to differentiation, the BT145 CICs were differentiated using media without EGF or FGF, but containing 10% FBS for 3 weeks. Following differentiation, the cytoskeletal stiffness, average traction stress, and percent of positive vinculin area were measured. The cytoskeletal stiffness of the differentiated BT145 (dBT145) tumor cells significantly increased to 1310 ± 90.0 Pa (p < 0.01), similar to that of the U87MG and A172 tumor cell lines (Fig. 1B). In addition, the cell traction forces of the dBT145s (29.2 ± 1.65 nN) were measured and the traction forces significantly increased (p < 0.01) compared to the primary BT145 tumor cells (Fig. 2). Finally, positive vinculin staining was quantified, and contrary to their non-differentiated counterparts, the dBT145 tumor cells adhered well onto the surface with observed stress fiber formation and had a similar percentage of positive vinculin area (7.93 ± 0.92%) as the U87MG and A172 tumor cells (Fig. 3). Moreover, cell morphologies between dBT145 tumor cells and their non-differentiated counterparts were observably distinct. After differentiation of the BT145 tumor cells, the cytoskeletal stiffness, average cell traction stress, and percent of positive vinculin area significantly increased compared to their undifferentiated CIC counterparts. In addition, these properties were similar to that of the less invasive cancer cell lines, U87MG and A172, supporting that differentiation of the cancer initiating cells leads to a less invasive phenotype. Therefore, this analysis of the biomechanical properties of the tumor cells supports previous reports that state CICs have a greater invasive potential than differentiated tumor cells^45^,^46^,^47^.

### Cytoskeletal Stiffness Analysis of Cells on Aligned Nanotopography Corroborates that Stiffness is Correlative of Invasive Potential

**Figure 4:**
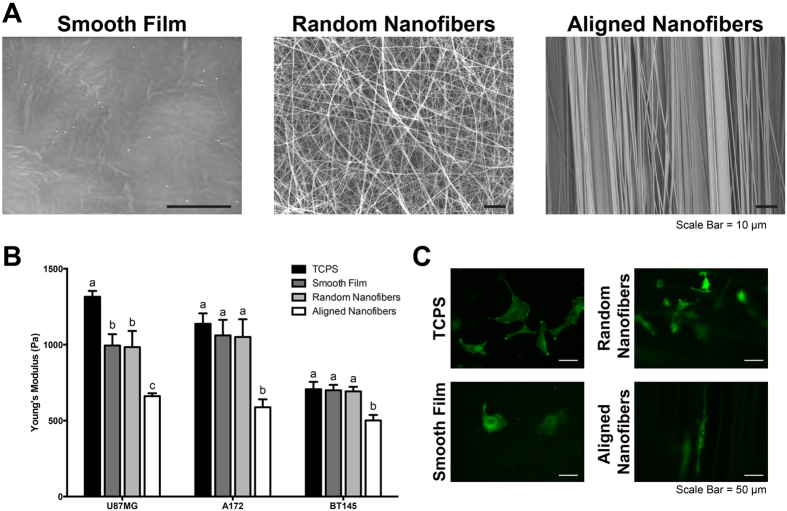
Aligned nanotopography resulted in decreased cytoskeletal stiffness. (**A**) Electrospinning was used to fabricate randomly aligned and aligned nanofibers of 668 ± 98.6 nm and 621 ± 173 nm, respectively. Lack of surface topography is observed in smooth film. (**B**) AFM was used to determine cytoskeletal stiffness of tumor cells when cultured on differing topographic substrates. When cultured on aligned nanofibers, cytoskeletal stiffness significantly decreased (p < 0.05) compared to each other substrate for each cell type. (**C**) Representative fluorescent images of A172-GFP tumor cells on different substrates. N = 3, mean ± SEM.

The measurement of cytoskeletal stiffness on a rigid 2D substrate provides important baseline characteristics of the cells; however it is important to measure these values on a scaffold that mimics the mechanical properties of the *in vivo* tumor microenvironment to obtain more biologically relevant results. Therefore, we used polycaprolactone (PCL) substrates (smooth film, randomly aligned nanofibers, and aligned nanofiber films) to investigate whether cell biomechanical properties would change. The nanofibers mimic the topographical cues provided by the white matter tracts and blood vessels. Electrospun nanofibers have been previously used as a model for glioblastoma and breast cancer migration^24^,^25^,^26^,^27^,^28^,^30^,^34^. By using a rotating mandrel, aligned nanofibers with an average diameter of 668 ± 98.6 nm were fabricated, which are within the range of diameters of white matter tracts observed in the human brain. While the diameters of these topographic substrates range from person to person (from 0.3 μm to 10 μm^48^), on average, median and average white matter tract diameters have been shown to be below 1 μm^49^. Randomly aligned nanofibers had an average diameter of 621 ± 173 nm. SEM images of the different substrates can be seen in Fig. 4A. The difference in alignment can be observed between the aligned and the randomly aligned nanofibers films. By using aligned nanofibers, randomly aligned nanofibers and a smooth, non-topographic film, we were able to investigate how topography and fiber orientation affected cell behavior.

In order to determine the effect of nanotopography on cytoskeletal stiffness, AFM was used to measure the cytoskeletal stiffness of U87MG, A172, and BT145 tumor cells seeded on collagen coated TCPS, smooth film, randomly aligned nanofibers, and aligned nanofibers (Fig. 4B). For both cell lines and CICs, the cytoskeletal stiffness was significantly lower on the aligned nanofibers (p < 0.05) compared to both randomly aligned nanofibers and smooth film with stiffness measurements of 995 ± 74.2 Pa, 984 ± 107 Pa, and 661 ± 18.5 Pa, for the U87MG cells on the smooth film, randomly aligned nanofibers, and aligned nanofibers, respectively. Stiffness for U87MG tumor cells cultured on the aligned nanofibers decreased 2-fold compared to TCPS and about 1.5-fold compared to the other substrates. Similar trends in stiffness were also observed for A172 and BT145 tumor cells. Representative fluorescent images of cells on each substrate can be seen in Fig. 4C.

It has been shown previously that cells cultured on aligned nanofibers migrate further and at a faster rate than on randomly aligned nanofibers or substrates without topography, similar to tumor cell migration speeds *in vivo*^24^,^25^,^26^. Combined with the data demonstrating that cytoskeletal stiffness is a biomarker for GBM invasive potential, we observe that the cells are more compliant when invasive potential is increased. The actin cytoskeleton is undergoing rapid depolymerization and polymerization during migration, which likely leads to the reduced stiffness^50^. In addition, Roca-Cusachs *et al.* investigated how the elongated cell shape affects the actin cytoskeleton and reduced cytoskeletal stiffness in endothelial cells^51^. They suggest that the spatial organization of the actin cytoskeleton of elongated cells contributes to the reduced cytoskeletal stiffness. The elongated cells lack the crosslinking between parallel actin networks that occurs in cells that are uniformly spread^51^. This data highlights that an aligned nanotopography has the ability to alter cytoskeletal stiffness, thereby promoting a more migratory cell state.

### Morphometric Analysis of Focal Adhesions Correlate Larger, Elongated Adhesions with a Migratory State

**Figure 5:**
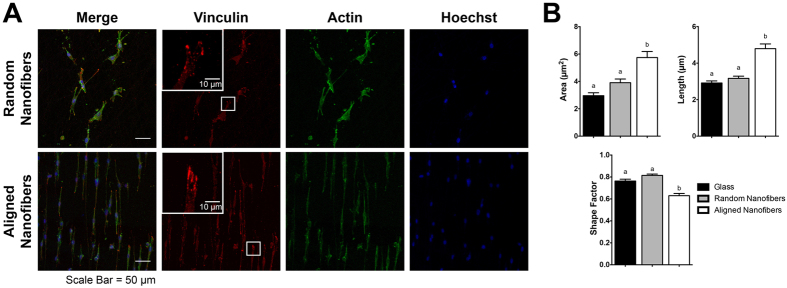
Focal adhesion analysis on glass, randomly aligned, and aligned nanofibers exhibits aligned nanotopography promotes larger and more elongated focal adhesions. (**A**) Representative images of U87MG tumor cells of vinculin (red), F-actin (green), and counterstained with Hoechst (blue) on aligned and randomly aligned nanofibers. White box highlights a magnified area in the inset for the fluorescent images showing vinculin staining. Aligned nanofibers promoted a spindled morphology, while randomly aligned nanofibers exhibited cells with multiple processes along the fiber directions. (**B**) Quantification and analysis of vinculin adhesions showed that cells on aligned nanofibers had adhesions that were significantly larger in area, longer, and more elliptical (p < 0.001) then when on randomly aligned fibers or glass. N = 21, mean ± SEM.

The cytoskeletal organization of cells on glass, randomly aligned nanofibers, and aligned nanofibers was analyzed to further describe the effect of topography on the cytoskeleton. As seen in Fig. 5, cells on the aligned nanofibers formed parallel actin filaments along the direction of the fibers. In addition, clusters of focal adhesions can be observed along the poles of these cells. Cells on randomly aligned fibers observed a more spread morphology, forming extensions in different directions along the fibers. Individual vinculin adhesions were characterized by area, length, and shape factor (Fig. 5B) as previously reported by Kim *et al*^52^. Focal adhesions on the aligned nanofibers were significantly larger in area and length than the adhesions on glass or randomly aligned nanofibers. Focal adhesion areas on aligned nanofibers, randomly aligned nanofibers, and glass were 5.74 ± 0.441 μm^2^, 3.91 ± 0.268 μm^2^, and 2.96 ± 0.209 μm^2^ respectively. In addition, adhesions also had a significantly smaller shape factor (4π(area)/(perimeter)^2^) on the aligned nanofibers (0.63 ± 0.020), indicating that the adhesions were more elongated and elliptically shaped than those cultured on glass (0.76 ± 0.17) and randomly aligned nanofibers (0.81 ± 0.012).

Substrate and ECM topography has an effect on stress fiber and focal adhesion formation. Human mesenchymal stem cells cultured on 500 nm gratings of TCPS exhibited increased vinculin protein expression compared to unpatterned controls. In addition, the cells on the topographic substrates exhibited a reduction in cytoskeletal stiffness and an elongated morphology with an aligned actin cytoskeleton and a dense focal adhesion population around the poles of the cells, compared to adhesions localized to both the central and peripheral region on cells on unpatterned substrates^10^. Similar results were found across a variety of cell types including epithelial, kidney, and fibroblast cells^53^,^54^,^55^. In addition, Kim *et al.* investigated the effects of individual focal adhesion size on cell motility in mouse embryonic fibroblasts. While a moderately correlative inverse linear relationship was found between shape factor and cell migration speed, they found that focal adhesion size was highly predictive of cell speed^52^. Similar results were also observed with C2C12 mouse myoblast cells cultured on aligned suspended nanofibers. These cells exhibited higher migration speeds, with focal adhesion clusters approximately 4x times longer than when grown on a smooth substrate^56^. An increased presence of longer filopodia along the leading edge has been observed in cells on an aligned nanotopography, and may aid in enhancing the migration speed^57^. Together, this suggests the aligned nanotopography provides the guidance cues necessary to reorganize the cytoskeleton to promote a propensity for a migratory state.

### Nanotopography and Alignment Correlates with Enhanced Migratory and Reduced Proliferative Phenotype in Tumor Cells

**Figure 6:**
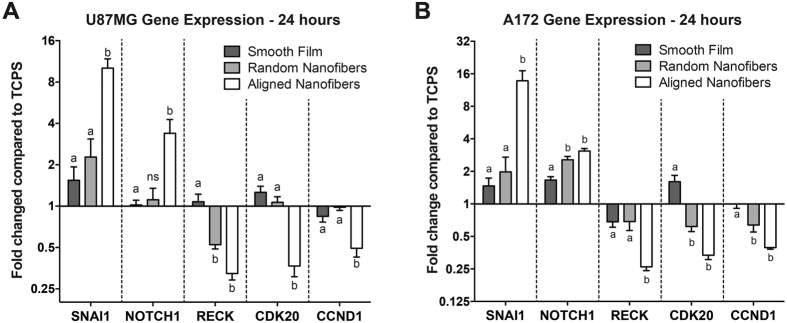
Aligned nanotopography resulted in the most significant increase in pro-migratory and decrease in pro-proliferative gene markers. Quantitative real time PCR for gene expression in U87MG (**A**) and A172 (**B**) cells when plated on TCPS, smooth film, randomly aligned nanofibers, and aligned nanofibers for 24 hours. Results are shown with fold expression relative to the TCPS condition. Aligned nanotopography resulted in the significant upregulation of pro-migratory marker SNAI1 and NOTCH1, downregulation of anti-migratory marker RECK, and decreased pro-proliferative markers CDK20 and CCND1 (p < 0.05). N = 3, mean ± SEM.

With the effect of topography on the biomechanical attributes of cells investigated, further analysis on gene expression along the migratory signaling pathways was completed using qRT-PCR. Gene expression analysis was completed for pro-migratory gene markers zinc finger protein SNAI1 (SNAI1) and Notch homolog 1 (NOTCH1), anti-migratory gene marker reversion-inducing-cysteine-rich protein with kazzal motifs (RECK), and pro-proliferative gene markers cyclin-dependent kinase 20 (CDK20) and cyclin D1 (CCND1). U87MG and A172 tumor cells exhibited greater than ±2 fold changes for all of the investigated genes when seeded on the aligned nanofibers compared to TCPS (Fig. 6). The fold increase on the aligned nanofibers compared to TCPS for pro-migratory SNAI1 and NOTCH1 markers was 10.12 and 3.39 for U87MG tumor cells, and 16.75 and 3.09 for A172 tumor cells, respectively. Anti-migratory RECK was downregulated 3.10 and 3.83 fold in the U87MG and A172 tumor cells, respectively. Pro-proliferative gene markers CDK20 and CCND1 were also downregulated 2.72 and 2.02 fold compared to TCPS for U87MG cells. Similar decreased gene expression was observed in A172 tumor cells, with 2.99 and 2.54 fold respective downregulation. Furthermore, there were statistical differences between the aligned nanofiber films and the smooth film substrate for all of the analyzed genes. When comparing the gene expression of the A172 tumor cells on aligned nanofibers and randomly aligned nanofibers for NOTCH1, a greater than 2-fold difference was observed. However, for the U87MG cells on the aligned nanofibers and randomly aligned nanofibers, a greater than 2-fold expression difference was observed for the expression of SNAI1.

Previous studies showed that culturing GBM tumor cells on aligned chitosan-PCL fibers of 200 and 400 nm significantly upregulated several migratory and EMT gene markers^26^. Our data demonstrated a significant increase in SNAI1 and NOTCH1 expression on the aligned nanofibers. Notch signaling has been shown to promote EMT through regulation of SNAI1^58^,^59^. Zhang *et al.* showed that elevation of NOTCH1 signaling in GBM cells and tumor biopsies led to increased tumor invasion and EMT markers expression^60^. Downregulation of metastasis suppressor RECK, which was observed on the aligned nanofibers, is essential for the invasiveness in GBM cells, as overexpression of RECK in T98G glioblastoma tumor cells altered the cytoskeleton to produce fewer lamellipodia and greater stress fibers, indicating decreased mobility^61^.

In addition, we also investigated the effect of substrate topography on genes responsible for proliferation. GBM tumors have exhibited a “go-or-grow” phenomenon in which there is a dichotomy between the migratory (go) and proliferative (grow) behavior of the tumor cells. Previous studies have shown that invading cells at the leading edge of the tumor have low proliferative index by inhibiting entry into the cell cycle, while cells located in the tumor core are highly proliferative, *in vitro* and *in vivo*^62^,^63^,^64^,^65^,^66^. Using an implantable nanofiber film to direct tumor cell migration, Jain *et al.* showed that cells migrating away from the tumor core were in a less proliferative state on the aligned nanofibers compared to a smooth film control, as suggested with the reduced Ki-67 staining. F-actin staining analysis showed that cells grown on the aligned nanofiber film exhibited uniform F-actin filaments compared to more punctate F-actin staining of cells on a smooth film, suggesting that the U87MG tumor cells were less migratory and in a suspended state on the smooth film^24^. Gene expression of CDK20 and CCND1, both of which play a role in the transition from the G1/S phase in the cell cycle^67^,^68^, were significantly downregulated on the aligned nanofibers, with a greater than 2-fold decrease compared to the TCPS control, further purporting that substrate nanotopography induces a propensity for a migratory GBM tumor cell state

## Conclusion

This study investigated the biomechanical properties of highly aggressive and migratory GBM tumor cells. We demonstrate that the cytoskeletal stiffness, traction stresses, and focal adhesion formation is significantly reduced in the highly invasive tumor cells compared to healthy astrocytes. In addition, by using an aligned nanofiber film that mimics the white matter tracts and blood vessels, we observed a reduction in cytoskeletal stiffness. Together, with the upregulation of migratory related genes on the aligned nanofibers, aligned actin cytoskeleton, and increased presence of larger, elongated adhesions, these data suggest the aligned nanotopography promotes the biophysical changes in the cells leading to an enhanced migratory state. Testing the biomechanical properties of GBM is an effective diagnostic methodology to determine the aggressiveness of GBM tumor. This *in vitro* model can further be applied to elucidate how the nanotopography of the substrates transduces into altered gene and protein expression to induce tumor cell invasion and migration, thus providing a mechanism to inhibit the process to form secondary tumor sites.

## Methods

### Cell Culture

Human GBM tumor cell lines, U87MG and A172 (ATCC, Manassas, VA), were maintained and cultured in DMEM (Corning, Corning, NY), supplemented with 10% fetal bovine serum (FBS, Gemini Bio-Products, West Sacramento, CA), 1% penicillin-streptomycin, 1% L-glutamine, and 1% non-essential amino acids (Corning). U87MG cells expressing enhanced green fluorescent protein (eGFP) were generously donated by Dr. Ravi Bellamkonda (Georgia Institute of Technology). A172 cells were transfected using GFP-Actin fusion lentiviral particles (GenTarget Inc., San Diego, CA) and positive colonies were selected for expansion. Primary CICs, BT145 were generously gifted by Dr. Rosalind Segal (Dana-Farber Cancer Institute). The GBM CICs were cultured in DMEM/F12 (Corning), supplemented with B27 (Gibco, Life Technologies Grand Island, NY), 15 mM HEPES (Alfa Aesar, Ward Hill, MA), 20 ng/mL EGF (Invitrogen, Carlsbad, CA), and 20 ng/mL FGF (Invitrogen). Differentiated BT145 tumor cells were grown in DMEM supplemented with 10% FBS. Primary rat astrocytes were isolated from Neonatal Sprague-Dawley pups (post-natal day 2) and maintained in Neurobasal Medium (Gibco), supplemented with 10% FBS and 1X GlutaMAX (Gibco). Mouse neural stem cells (Cyagen) were maintained and cultured in NeuroCult™ NSC Basal Medium (STEMCELL Technologies) supplemented with 1% penicillin-streptomycin, B27, 5 μg/mL FGF, 10 μg/mL EGF, and 0.2% v/v heparin.

### Preparation of Nanotopographic Scaffolds

Aligned and randomly aligned nanofibers were fabricated using electrospinning as previously described^69^. Briefly, a 10% polycaprolactone (w/v, PCL, Sigma, St. Louis, MO) was dissolved in 1,1,1,3,3,3-hexafluoro-2-propanol (HFIP, Sigma) overnight. To fabricate aligned nanofibers with a diameter of 600–800 nm, a 19 gauge blunt tipped needle was used to eject the polymer jet and was collected on a spinning mandrel (3000 RPM) 10 cm away for 20 min to create a film of 10 μm thickness. A positive voltage (5–8 kV) was applied (Model ES30P, Gamma High Voltage Research). To fabricate randomly aligned nanofibers, the flow rate was adjusted to 0.5 mL/hr, charged with 8 kV, and collected for 10 minutes onto the mandrel spinning at 15 RPM. To fabricate a smooth film substrate, 12% PCL was spread onto a glass coverslip and the HFIP was evaporated under vacuum. Smooth film and TCPS were used as non-topographic controls.

### Characterization of Substrates

To characterize the substrates, SEM was performed on the aligned nanofibers, randomly aligned nanofibers, and smooth film substrates as previously described^24^. Briefly, samples were mounted onto stubs using carbon tape. Smooth film SEM images were taken using a FEI-TeneoLoVac SEM and aligned and randomly aligned nanofiber films were taken using a JSM-7000F SEM. Fiber diameter analysis was completed using ImageJ.

### Coating of Topographical Substrates

Substrates were coated with PureCol^®^ Bovine Collagen (100 μg/mL, Advanced BioMatrix, San Diego, CA) prior to culturing the cells to aid in cellular adhesion. The collagen was added onto of the substrates and allowed to incubate at 37 °C for 1–2 hours. The substrates were then washed 3 times with sterile phosphate buffered saline (PBS) prior to seeding the cells. For coating with laminin, substrates were pre-coated with poly-L-lysine (Sigma) for 30 minutes, before being rinsed with sterile water, and coated with 50 μg/mL laminin (50 μg/mL, Invitrogen) overnight at 37 °C.

### Cytoskeletal Force Measurements

To measure the cytoskeletal stiffness measurements, tumor cells were seeded on the collagen-coated tissue culture polystyrene (TCPS) or PCL substrates at a density of 20,000 cells per sample. The tumor cells were cultured for 24 hours prior to testing. An MPF3D-Bio AFM (Asylum Research, Santa Barbara, CA) was used to measure the stiffness of cells as previous described^70^. Briefly, the DNP-D cantilevers were calibrated using the thermal method prior to experiment. A Nikon Ti-S microscope (MVI, Avon, MA), was used to observe and select the desired cells for measurement. The AFM cantilever was placed over the perinuclear region of the cell, and an indentation was performed with a maximum cantilever deflection of 10 nm. After maximum deflection, the z-position of the AFM scanner was oscillated with a frequency of 10 Hz, amplitude of 25 nm, and duration of 1.5 seconds. The AFM tip was removed from the cell, and the z-position of the tip and cantilever deflection was recorded. A total of 3 measurements were obtained at different locations in the perinuclear region of the cell, a minimum of 15 cells was measured for each sample, and each sample was tested in biological triplicates. The Hertz indentation model was used to calculate the stiffness of each cell using a custom MATLAB (MathWorks, Natick, MA) code.

Traction force measurements were determined by culturing the astrocytes or tumor cells on polyacrylamide gels with a thin layer of fluorescent beads embedded within them. Briefly, FluoSpheres (Life Technologies) were embedded into a gel comprised of 5% or 8% acrylamide and 0.08% or 0.1% bis-acrylamide, respectively. Prior to plating, the gel was coated and crosslinked with PureCol^®^ Bovine Collagen (100 μg/mL) overnight using Sulfo-SANPAH (Pierce). After coating, 10,000 astrocytes or tumor cells were plated onto the gels. After 24 hours, fluorescent images of the spheres and brightfield images of cell on the stressed gel were taken using an Olympus IX83 inverted microscope. Following this, media was removed and 0.25% trypsin EDTA (Corning) was added to release the cell from the substrate, returning the gel to a relaxed state. Corresponding images of the beads and gel (without the cell) were taken at the same location. A minimum of 7 cells was analyzed per gel, with each gel being repeated in triplicate. Traction forces were calculated using MATLAB and ANSYS software (Ansys Inc., Canonsburg, PA). Traction forces were normalized to respective cell area to determine average cell traction stress.

### Immunocytochemistry and Analysis

To stain the astrocytes and tumor cell cultures with vinculin and F-actin, approximately 15,000 tumor cells or astrocytes were cultured on collagen-coated substrates overnight (about 24 hours) before being briefly rinsed with cytoskeleton buffer. Following this, cells were fixed with 4% paraformaldehyde for 10 minutes, permeabilized with 0.1% Triton-X-100 (Sigma) for 20 minutes, and blocked in 4% goat serum for 30 minutes. Cells were then stained for focal adhesion protein vinculin (1:500, V9131, Sigma), F-actin with phalloidin (Life Technologies), and counterstained with Hoechst 33342 (Life Technologies). A Leica SP5 Point Scanning Confocal microscope was used to image the cytoskeletal organization on the substrates. Morphometric analysis of the focal adhesions of the GBM tumor cells were conducted on the images using ImageJ software. Briefly, an ellipse outline was drawn on each individual focal adhesion. The length, area, and shape factor were then determined based on the focal adhesion’s shape. An average of each shape descriptor was calculated for each cell. A minimum of 7 cells was analyzed for each substrate sample, and 3 substrate samples were analyzed for each condition.

### Analysis of Key Migratory and Proliferative Gene Markers

To analyze key migratory and proliferative gene makers, quantitative reverse transcription polymerase chain reaction (qRT-PCR) was performed. In order to collect the mRNA, 750,000 tumor cells were seeded onto each substrate. Twenty-four hours post-seeding, the media was removed and RLT Buffer (Qiagen, Venlo, Netherlands) was added to the cultures for 20 minutes to lyse cells. The solution was then collected, processed, and purified using the RNeasy kit (Qiagen). The mRNA concentration for each sample was measured at the 260/280 absorbance using NanoDrop 2000 spectrophotometer (Thermo Fisher Scientific). The cDNA was made using the equivalent of 1 μg of RNA and iScript cDNA synthesis kit (Bio-Rad, Hercules, CA). Migratory markers, zinc finger protein SNAI1 (SNAI1), Notch homolog 1 (NOTCH1), and reversion-inducing-cysteine-rich protein with kazzal motifs (RECK), and proliferative markers, cyclin-dependent kinase 20 (CDK20), and cyclin D1 (CCND1), were probed using the iTaq SYBR Green Supermix (Bio-Rad). For analysis of each gene, and the values were normalized to the housekeeping gene GAPDH. Primer sequences are listed in Table 1.

### Statistical Analysis

All statistical analysis was performed by using GraphPad Prism (GraphPad Software, San Diego, CA). Data was reported as mean ± standard error of the mean (SEM), and either a t-test or a one-way ANOVA with Tukey’s post-hoc analysis was performed to determine differences amongst substrates. Data were considered statistically significant for a p value < 0.05.

## Additional Information

**How to cite this article**: Beliveau, A. *et al.* Aligned Nanotopography Promotes a Migratory State in Glioblastoma Multiforme Tumor Cells. *Sci. Rep.* **6**, 26143; doi: 10.1038/srep26143 (2016).

## Figures and Tables

**Table 1:** Forward and Reverse Primers.

Gene	Forward Primer	Reverse Primer
GAPDH	5′-TGTAGTTGAGGTCAATGAAGGG-3′	5′-ACATCGCTCAGACACCATG-3′
SNAI1	5′-GGTTCTTCTGCGCTACTGC-3′	5′-GCTGGAAGGTAAACTCTGGATT-3
NOTCH1	5′-CAGGCAATCCGAGGACTATG-3′	5′-CAGGCGTGTTGTTCTCACAG-3
RECK	5′-CAATAGCCAGTTCACAGCAG-3′	5′-CCAGATTATTGCCCAGAGACA-3′
CDK20	5′-CAGTGTCTGCCTTCTATCCTG-3′	5′-TGTACCACATACTGATTGTCCTC-3′
CCND1	5′-GCCCTCGGTGTCCTACTTC-3′	5′-CTGTTCCTCGCAGACCTCC-3′
